# 1214. Short versus long antibiotic treatment duration for febrile urinary tract infection and acute pyelonephritis: A systematic review and meta-analysis

**DOI:** 10.1093/ofid/ofad500.1054

**Published:** 2023-11-27

**Authors:** Erika Schneider-Smith, Bailey Bacher, Xavier Morgan-Gillard, Lauren Yaeger, Michael J Durkin, Jonas Marschall

**Affiliations:** Washington University in St. Louis, St. Louis, Missouri; Washington University in St. Louis, St. Louis, Missouri; Washington University in St. Louis, St. Louis, Missouri; Washington University School of Medicine in St. Louis, St. Louis, Missouri; Washington University School of Medicine, St. Louis, Missouri; Washington University School of Medicine in St. Louis, St. Louis, Missouri

## Abstract

**Background:**

The optimal treatment duration for febrile UTI and pyelonephritis is unknown; however, multiple randomized trials have compared shorter versus longer duration. The most recent systematic review and meta-analysis was published 10 years ago.

**Methods:**

With the support of a medical librarian (LY), we conducted a systematic review of the pertinent literature up to December 14, 2021. We used key terms for febrile urinary tract infection and acute pyelonephritis, treatment duration, and possible antimicrobial agents in the search. Using the evaluation tool Covidence, the identified abstracts were reviewed by the research team and potentially eligible studies were subject to full-text review. We also reviewed the references of eligible articles and scrutinized meeting abstracts. For the meta-analysis, we used SPSS version 28. For quality assessment, we used the Cochrane risk-of-bias tool.

**Results:**

We screened 6732 abstracts, reviewed 21 full-text articles, and included 12 articles in this systematic review. We conducted a meta-analysis of these 12 articles (Figure 1). The Odds Ratio (OR) for clinical failure between short versus long treatment duration was 0.81 (95% CI 0.55-1.19). We also conducted a subgroup analysis for the more recent trials (#9-12 in Fig 1), with an OR 1.09 (95% CI 0.64-1.87), and the older trials (#1-8 in Fig 1), with an OR 0.68 (95% CI 0.36-1.31). The OR of experiencing an adverse event was 0.88 (95% CI 0.63-1.22) between short and long duration groups (Fig 2).

**Figure 1:**
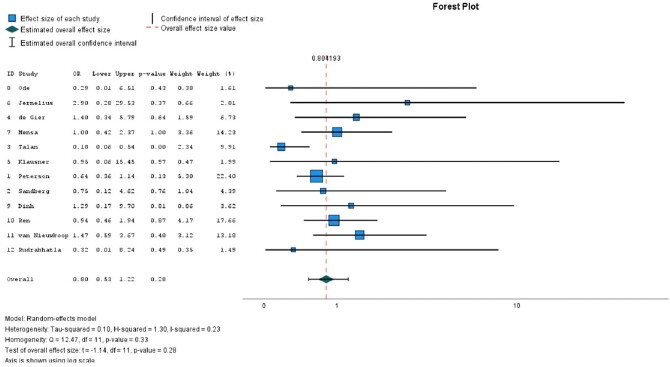
Odds ratio of treatment failure with short versus long antibiotic course for febrile UTI and acute pyelonephritis

This figure represents the odds ratio of treatment failure in short versus long duration treatment. Therefore, an odds ratio <1 favors short duration (i.e. lower odds of treatment failure), whereas an odds ratio >1 favors long duration.

**Figure 2:**
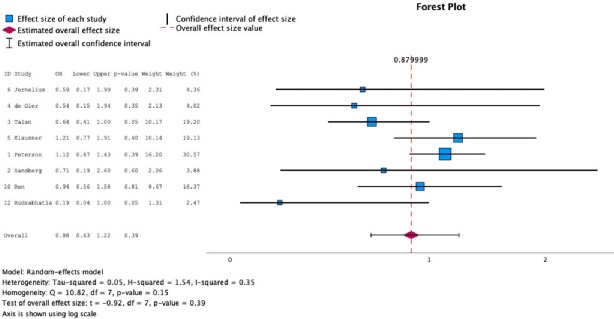
Odds ratio of adverse events with short versus long antibiotic course for febrile UTI and acute pyelonephritis

This figure represents the odds ratio of a patient experiencing at least one adverse event in the short versus long duration treatment groups. Therefore, an odds ratio <1 favors short duration (i.e. lower odds of adverse events), whereas an odds ratio >1 favors long duration.

**Conclusion:**

In this systematic review and meta-analysis of the evidence comparing shorter versus longer treatment durations for febrile UTI and pyelonephritis, 7 days of treatment were non-inferior to 14 days. There was no difference in adverse events between the two groups. Our findings confirm those of an earlier meta-analysis and serve as a reminder that shorter treatment durations for febrile UTI and pyelonephritis are sufficient.

**Disclosures:**

**All Authors**: No reported disclosures

